# Proteome analysis of shell matrix proteins in the brachiopod *Laqueus rubellus*

**DOI:** 10.1186/s12953-015-0077-2

**Published:** 2015-08-15

**Authors:** Yukinobu Isowa, Isao Sarashina, Kenshiro Oshima, Keiji Kito, Masahira Hattori, Kazuyoshi Endo

**Affiliations:** Department of Earth and Planetary Science, Graduate School of Science, The University of Tokyo, 7-3-1 Hongo, Bunkyo-ku, Tokyo, 113-0033 Japan; The University Museum, The University of Tokyo, 7-3-1 Hongo, Bunkyo-ku, Tokyo, 113-0033 Japan; Center for Omics and Bioinformatics, Department of Computational Biology, Graduate School of Frontier Sciences, The University of Tokyo, 5-1-5 Kashiwanoha, Kashiwa, Chiba 277-8561 Japan; Department of Life Science, School of Agriculture, Meiji University, 1-1-1 Higashimita, Tama, Kawasaki, Kanagawa 214-8571 Japan

**Keywords:** Brachiopoda, Biomineralization, Shell matrix protein, Proteome, Transcriptome

## Abstract

**Background:**

The calcitic brachipod shells contain proteins that play pivotal roles in shell formation and are important in understanding the evolution of biomineralization. Here, we performed a large-scale exploration of shell matrix proteins in the brachiopod *Laqueus rubellus*.

**Results:**

A total of 40 proteins from the shell were identified. Apart from five proteins, i.e., ICP-1, MSP130, a cysteine protease, a superoxide dismutase, and actin, all other proteins identified had no homologues in public databases. Among these unknown proteins, one shell matrix protein was identified with a domain architecture that includes a NAD(P) binding domain, an ABC-type transport system, a transmembrane region, and an aspartic acid rich region, which has not been detected in other biominerals. We also identified pectin lyase-like, trypsin inhibitor, and saposin B functional domains in the amino acid sequences of the shell matrix proteins. The repertoire of brachiopod shell matrix proteins also contains two basic amino acid-rich proteins and proteins that have a variety of repeat sequences.

**Conclusions:**

Our study suggests an independent origin and unique mechanisms for brachiopod shell formation.

**Electronic supplementary material:**

The online version of this article (doi:10.1186/s12953-015-0077-2) contains supplementary material, which is available to authorized users.

## Background

Many organisms have a diversity of biominerals that have a large number of ecologically important functions, including body support and defense against predators. Most metazoan biominerals are likely acquired independently in different phyla in the early Cambrian [1]. On the other hand, presence of some biomineralization genes shared by several phyla suggests that at least a part of the mechanisms was derived from a common ancestor [2]. A crucial factor in understanding the mechanisms and evolution of biomineralization is an in-depth analysis of the organic matrix present in hard tissues. A large number of skeletal proteins have been identified and their roles in the biomineralization process have been analyzed [3]. Recently, it became possible to identify skeletal proteins comprehensively using transcriptome analysis combined with proteome analysis across a wide range of phyla [4–9]. Brachiopods are marine invertebrates that appeared in the Cambrian and have two valves composed of calcium carbonate or calcium phosphate. Organic shell matrices in brachiopods have been studied in some detail [10, 11]. Previous SDS-PAGE analyses showed several major bands stained by Coomassie Brilliant Blue (CBB) [10, 12], and an amino acid composition analysis revealed that glycine and alanine account for a large proportion of bulk shell extracts [13, 14]. In addition to these studies, shell matrix proteins of brachiopods have been studied for the characterization of peptides and amino acids preserved in fossil brachiopod shells and immunological assays showed that some peptide sequences in the shells are preserved for about 1 million years [15, 16]. However, the amino acid sequences of the shell matrix proteins in brachiopods have not been identified, except for the partial amino acid sequencing of a chromoprotein, named ICP-1 [17–19]. In this study, we performed a large-scale analysis of shell matrix proteins of the brachiopod *Laqueus rubellus* using proteomics combined with transcriptomics. The analysis identified 40 shell matrix proteins. These new proteome data contained 35 protein sequences that have no database-related homologues and indicate that the mechanism and evolution of brachiopod shell formation are unique.

## Results

**Fig. 1:**
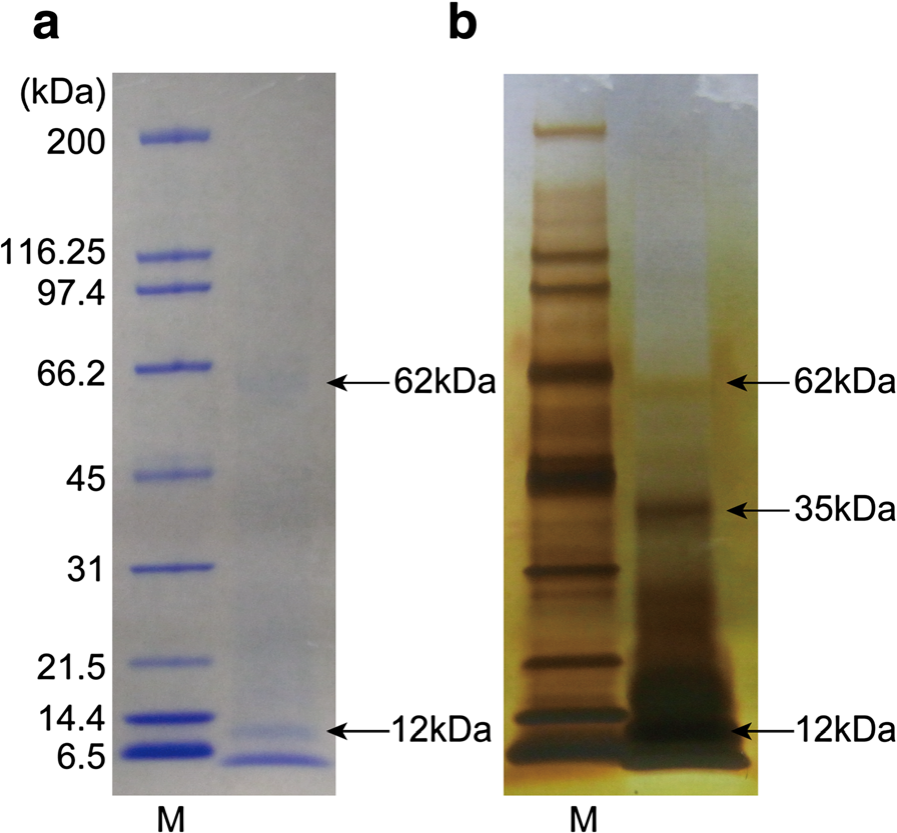
EDTA-soluble extracts from shell secondary layer of *Laqueus rubellus* were fractionated by SDS-PAGE. **a**: CBB **b**: silver staining M: marker

**Fig. 2:**
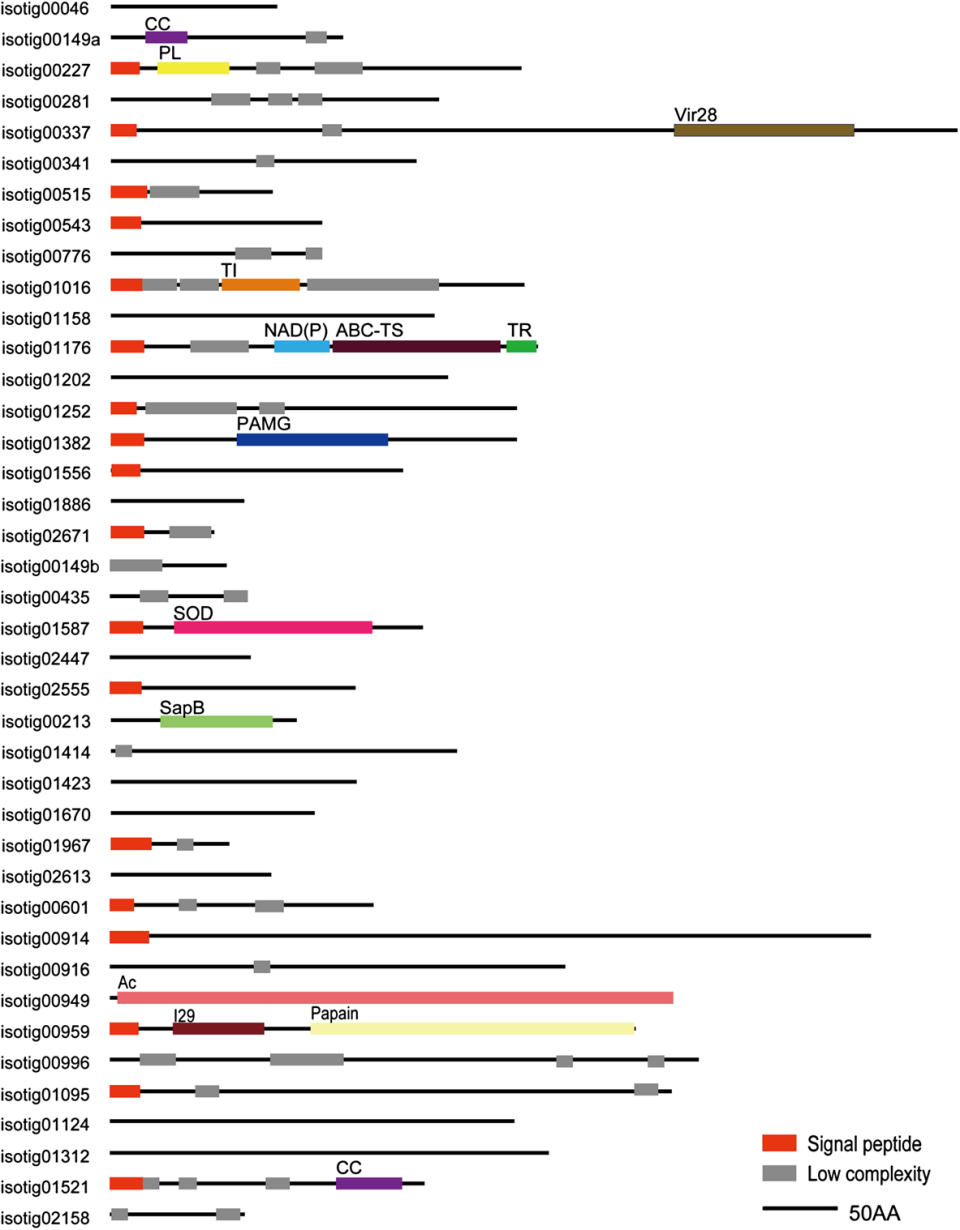
Schematic of the domains in shell matrix proteins identified in this study. CC: coiled coil; PL: pectin lyase-like; Vir28: variable surface protein Vir28; TI: trypsin inhibitor-like cysteine-rich domain; NAD(P): NAD(P)-binding Rossmann-fold domains; ABC-TS: ABC-type transport system; TR: transmembrane region; PAMG: Pneumovirinae attachment membrane glycoprotein G; SOD: copper/zinc superoxide dismutase; SapB: saposin B domains; Ac: Actin; I29: cathepsin propeptide inhibitor domain; and Papain: papain family cysteine protease

**Fig. 3:**
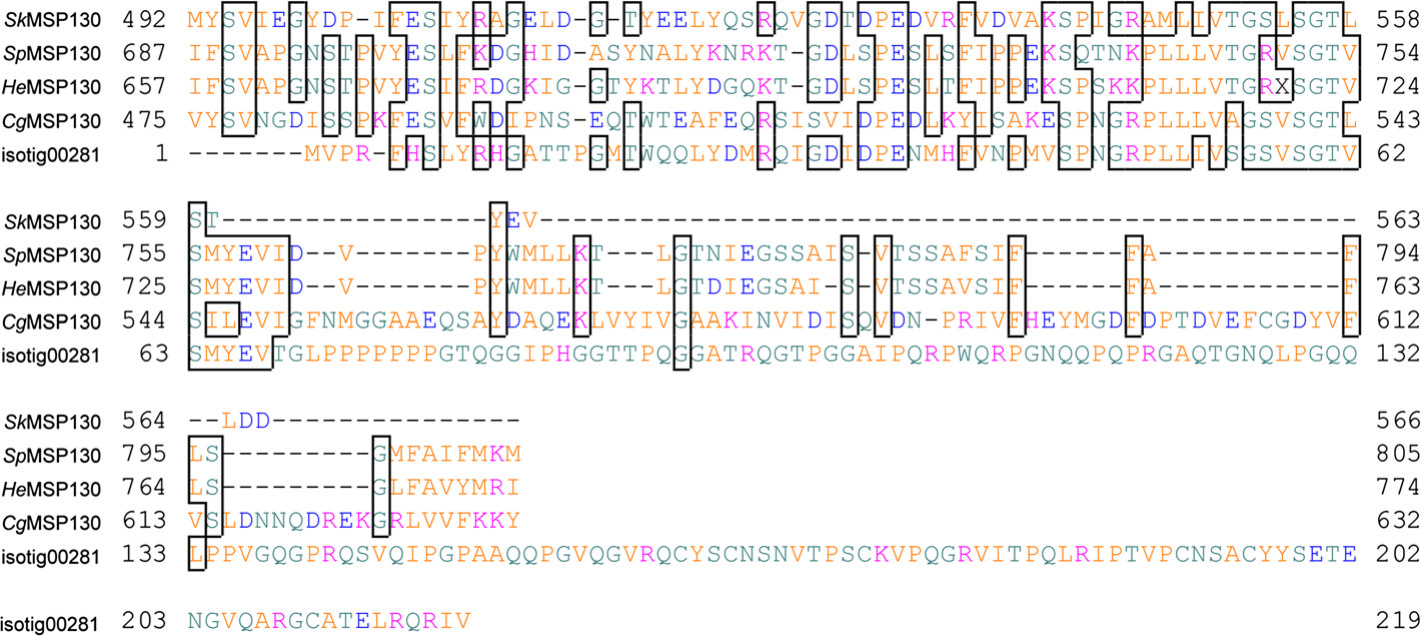
Alignment of the amino acid sequences of MSP-130 and isotig 00281. Sk: *Saccoglossus kowalevskii* (NCBI Acc. No. XP_002739468.1); Sp: *Strongylocentrotus purpuratus* (NCBI Acc. No. NP_001116986.1); He: *Heliocidaris erythrogramma* (NCBI Acc. No. CAC20358.1); and Cg: *Crassostrea gigas* (NCBI Acc. No. EKC20477.1)

**Fig. 4:**
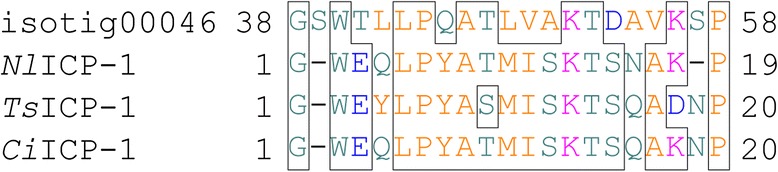
Alignment of the amino acid sequences of ICP-1 and isotig 00046. Nl: *Neothyris lenticularis*; Ts: *Terebratella sanguinea*; and Ci: *Calloria inconspicua*

**Fig. 5:**
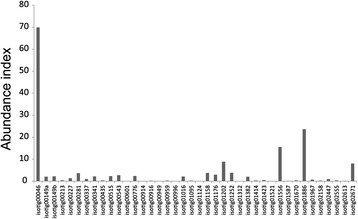
Abundance index of shell matrix proteins identified in this study

SDS-PAGE of the soluble organic matrix extracted from the secondary layer showed two major bands (< 6.5 and 12 kDa) and a minor band of 62 kDa when stained with CBB (Fig. 1a). Silver staining showed a major band at 35 kDa and many minor bands in addition to the three bands stained with CBB (Fig. 1b). Transcriptome sequencing in mantle tissue generated a total of 125,437 reads. To identify shell matrix proteins from *Laqueus rubellus*, we performed a proteome analysis of the material from these different sources, namely (1) the soluble organic matrix from the whole shell, (2) insoluble organic matrix from the whole shell and (3) soluble organic matrix from the shell secondary layer. The generated MS/MS data were subjected to SEQUEST searches against the protein sequence database obtained from the transcriptome analysis. As a result, we identified 40 shell matrix proteins (Tables 1, 2, 3, 4 and Fig. 2). Among these proteins, 18 proteins were identified from the soluble organic matrix of the shell secondary layer, and the soluble and insoluble organic matrix from the whole shell (Table 1). While five proteins were identified from both the soluble and insoluble organic matrix of the whole shell, 17 proteins were only identified from either the soluble or insoluble organic matrices of the whole shell (Tables 2, 3, 4). A total of 22 proteins were deduced to be possibly complete sequences, because these sequences have a stop codon and either an in-frame start codon just after an in-frame stop codon in the 5′ region of the sequence or a potential signal peptide (Tables 1, 2, 3, 4). A blast search against the GenBank non-redundant database showed that 36 out of the 40 shell matrix proteins have no annotated homologous sequences. Among the proteins that have sequence homology, isotig 00281 showed relatively high sequence similarity with MSP130, which is a skeletal protein identified from sea urchins (Fig. 3) [20, 21]. Isotig 01587, isotig 00949, and isotig 00959 showed high sequence similarity with extracellular copper/zinc superoxide dismutase, actin I, and cathepsin L cysteine proteinase, respectively (Tables 2 and 4). Predicted molecular mass and isoelectric point are shown in Tables 1, 2, 3, 4, and domains predicted by the SMART program and a National Center for Biotechnology Information (NCBI) conserved domain search are schematically shown in Fig. 2. Using the known amino acid sequences of ICP-1 [19], we performed local blast searches against sequence data obtained from this study. As a result, isotig 00046 was found to have a high sequence similarity with ICP-1 (Fig. 4). Gene expression analysis showed that ICP-1 gene is expressed in lophophore as well as in mantle tissues (Additional file 1). To estimate the abundance of each protein, we calculated the relative copy number based on the identified spectral counting [22–25]. The number of identified spectra of each protein was divided by the number of theoretically observable tryptic peptide ions, which have a mass-to-charge ratio of 400 to 1500 at two or three charge states, to generate an abundance index as the relative copy number. The result showed that isotig 00046 (ICP-1) is the most abundant protein in the shell extracts among the proteins identified in this study (Fig. 5). The percentage of spectra that were matched to peptide contained in protein sequences translated from transcriptome data was about 10 % of the total MS/MS spectra acquired. The proportion in this case was lower than those of other mass spectrometric analyses for organisms of which genome has been completely sequenced (*S. cerevisiae*, 40-50 %), suggesting presence of many more proteins in shell matrix samples than proteins identified in this study. Messenger RNA sequences in the transcriptome data may not include complete list for proteins in shell matrix or unknown chemical modifications that were not considered in our database search may occur in a large proportion of shell matrix proteins. The depth of isotigs is shown in Additional file 2.

**Table 1:** Shell matrix proteins identified from the whole shell and shell secondary layer

Isotig no.	Accession no.	Matching peptides	Complete sequence	Blast hit (e-value <1e^−10^)	Molecular mass (kDa)	pI
Isotig 00046	FX982984	11	−	−	−	−
Isotig 00149a	FX982985	4	○	−	16.9	10.1
Isotig 00227	FX982987	4	○	−	27.0	11.6
Isotig 00281	FX982988	7	−	MSP130	−	−
Isotig 00337	FX982989	6	○	−	58.0	10.30
Isotig 00341	FX982990	5	−	−	−	−
Isotig 00515	FX982992	4	○	−	9.2	9.38
Isotig 00543	FX982993	4	○	−	13.4	5.24
Isotig 00776	FX982995	4	−	−	−	−
Isotig 01016	FX983001	8	○	−	27.3	8.63
Isotig 01158	FX983004	6	−	−	−	−
Isotig 01176	FX983005	6	○	−	29.4	4.16
Isotig 01202	FX983006	11	○	−	25.0	4.68
Isotig 01252	FX983007	7	○	−	28.9	9.68
Isotig 01382	FX983009	5	−	−	−	−
Isotig 01556	FX983013	8	○	−	19.9	8.96
Isotig 01886	FX983016	6	○	−	10.0	8.47
Isotig 02671	FX983022	2	○	−	5.0	7.14

‘-‘means not applicable

**Table 2:** Shell matrix proteins identified from the whole shell (the soluble and insoluble organic matrix)

Isotig no.	Accession no.	Matching peptides	Complete sequence	Blast hit (e-value <1e^−10^)	Molecular mass (kDa)	pI
Isotig 00149b	FX983023	6	−	−	−	−
Isotig 00435	FX982991	2	○	−	9.1	5.95
Isotig 01587	FX983014	2	○	Extracellular copper/zinc Superoxide dismutase	22.4	8.26
Isotig 02447	FX983019	3	−	−	−	−
Isotig 02555	FX983020	2	−	−	−	−

‘-‘means not applicable

**Table 3:** Shell matrix proteins identified from the whole shell (the soluble organic matrix)

Isotig no.	Accession no.	Matching peptides	Complete sequence	Blast hit (e-value <1e^−10^)	Molecular mass (kDa)	pI
Isotig 00213	FX982986	3	−	−	−	−
Isotig 01414	FX983010	3	○	−	26.2	8.53
Isotig 01423	FX983011	3	○	−	18.1	10.58
Isotig 01670	FX983015	3	−	−	−	−
Isotig 01967	FX983017	2	○	−	8.7	6.09
Isotig 02613	FX983021	2	−	−	−	−

‘-‘means not applicable

**Table 4:** Shell matrix proteins identified from the whole shell (the insoluble organic matrix)

Isotig no.	Accession no.	Matching peptides	Complete sequence	Blast hit (e-value <1e^−10^)	Molecular mass (kDa)	pI
Isotig 00601	FX982994	2	−	Hypothetical protein	19.3	8.48
Isotig 00914	FX982996	2	○	Predicted protein	57.6	9.69
Isotig 00916	FX982997	2	−	−	−	−
Isotig 00949	FX982998	2	○	Actin I	41.7	5.18
Isotig 00959	FX982999	3	○	Cathepsin L cysteine proteinase	39.8	6.87
Isotig 00996	FX983000	2	−	−	−	−
Isotig 01095	FX983002	4	○	−	41.3	9.76
Isotig 01124	FX983003	2	−	Hypothetical protein	−	−
Isotig 01312	FX983008	2	−	Hypothetical protein	−	−
Isotig 01521	FX983012	2	○	−	25.1	11.85
Isotig 02158	FX983018	2	−	−	−	−

‘-‘means not applicable

## Discussion

### Repertoire of matrix proteins found in brachiopod shells

#### ICP-1

ICP-1 (IntraCrystalline Protein-1) is a shell matrix protein extract from the calcitic shell of three brachiopod species: *Neothyris lenticularis*, *Calloria inconspicua*, and *Terebratella sanguinea*. The partial N-terminal amino acid sequences of ICP-1 from these species have been determined by Edman degradation [17–19]. The local blast searches showed that the partial sequences of ICP-1 have a sequence similarity with a part of isotig 00046 (Fig. 4). Isotig 00046 showed the highest abundance in the shell among the shell matrix proteins identified in this study (Fig. 5). This is consistent with the results of a previous study [19]. ICP-1 was originally identified as a 6.5-kDa major band revealed by SDS-PAGE analysis of shell extracts from *Neothyris lenticularis*, *Calloria inconspicua*, and *Terebratella sanguinea* [19]. The predicted molecular mass of isotig 00046 is just over 11.9 kDa, while the thickest band in the SDS-PAGE analysis of *Laqueus rubellus* is < 6.5 kDa (Fig. 1). One possibility to explain this size discrepancy is that ICP-1 undergoes proteolytic cleavage after translation. We have identified cathepsin L cysteine proteinase from the shell extracts. This protein could be involved in post-translational modification. HPLC analysis in a previous study indicated that carotenoids are bound to ICP-1 [19]. The observation that ICP-1 shows the highest abundance in the shell suggests that ICP-1 plays key roles in the biomineralization processes in brachiopods. RT-PCR analysis showed that ICP-1 gene is also expressed in the lophophore tissues, suggesting that ICP-1 is also involved in the formation of the calcareous loop structure embraced by the lophophore.

#### MSP130

Isotig 00281 showed relatively high sequence similarity with MSP130 (The e-value against MSP130 from *Saccoglossus kowalevskii* was 1e^−10^). MSP130 was originally identified from primary mesenchyme cells in the sea urchin [20, 21] and was subsequently detected in the hard tissues of sea urchins [26, 27]. Homologues and closely related proteins of MSP130 were also reported to be present in molluscan shells [28]. In addition, MSP130 have been found in genomes of hemichordate, cephalochordate, bacteria, and green algae [29]. MSP130 is predicted to have been acquired by independent horizontal gene transfer in Cambrian, because this gene exists in bacteria and has an extremely wide phylogenetic distribution [29]. However, it appears possible that MSP130 gene was already present in the metazoan or bilaterian last common ancestor because the phylogenetic tree of MSP130 constructed in the previous study was divided into two clusters of the bacterial/green algae clade and the metazoan (bilaterian) clade [29]. If the MSP130 gene was transferred to animals horizontally many times independently, the animal MSP130 genes would not form a monophyletic cluster. The functions of MSP130 have not been determined, but this protein is predicted to function at the cell surface [30]. Identification of MSP130 from the brachiopod shells in addition to the skeletons of sea urchin and molluscs suggests that this protein plays an important role in biomineralization processes.

#### Digestive enzymes and inhibitors

Many digestive enzymes have been identified from the shell of *Laqueus rubellus*. Isotig 00227 has a pectin lyase-like domain, which enzymatically breaks down pectin. Pectin is usually found in plant cells, but some neutral sugars have been detected in terebratulide brachiopod shells [12]. Thus, isotig 00227 could be involved in the breakdown of these neutral sugars. Two lysosomal proteins were identified from the shell: (i) isotig 00213 is a saposin protein that is involved in lipid degradation and (ii) isotig 00959 is cathepsin L, which is a cysteine protease that plays a major role in intracellular protein catabolism. However, a trypsin inhibitor domain was found in the amino acid sequence of isotig 01016. A number of protease inhibitors have been identified from molluscan shells and a possible function of these proteins could be to protect the shell matrix proteins against particular proteases [31, 32]. In brachiopod shell formation, a protease inhibitor could play a similar role, but since secreted proteases and protease inhibitors are involved in modification of extracellular and membrane bound proteins in many systems, their roles may not be specific to shell matrix modifications.

#### Membrane protein

Isotig 01176 has a transmembrane region, indicating that this protein binds to the cell membranes of the mantle tissue. Interestingly, several skeletal proteins of a coral also have a transmembrane region [8]. If this protein is involved in shell formation, the mantle epithelium would be closely attached to the inner shell surface when mineralization occurs. Isotig 01176 also has a NAD(P) binding domain. NADP is a hydrogen carrier that is used in metabolic pathways such as the photosynthetic pathway and glycolysis as a reducing agent. Although the function of this domain in isotig 01176 is unknown, the high importance of H^+^ in CaCO_3_ synthesis suggests that the NAD(P) binding domain of isotig 01176 could function to sequester H^+^ from the crystallization milieu. Isotig 01176 may also have an ABC-type transport system, which is involved in the transportation of many types of substrates. This suggests that isotig 01176 could also control ion concentrations in the space where crystallization occurs. As isotig 01176 has all these functional domains, this protein is likely to have key roles in brachiopod shell formation.

#### Secreted protein

Among the 40 shell matrix proteins, 18 have a signal peptide, indicating that many shell matrix proteins are secreted from cells. Generating the calcium carbonate in the extracellular space is consistent with brachiopod shells being an exoskeleton. However, seven proteins do not have a signal peptide even though they have complete sequences. One possibility is that these proteins bind with other proteins that have a signal peptide. Actin, which is one of the major intracellular proteins, was identified. However, there is a possibility that this protein is occluded from trapping of proteins involved in the secretory processes.

### Amino acid composition and isoelectric points

**Fig. 6:**
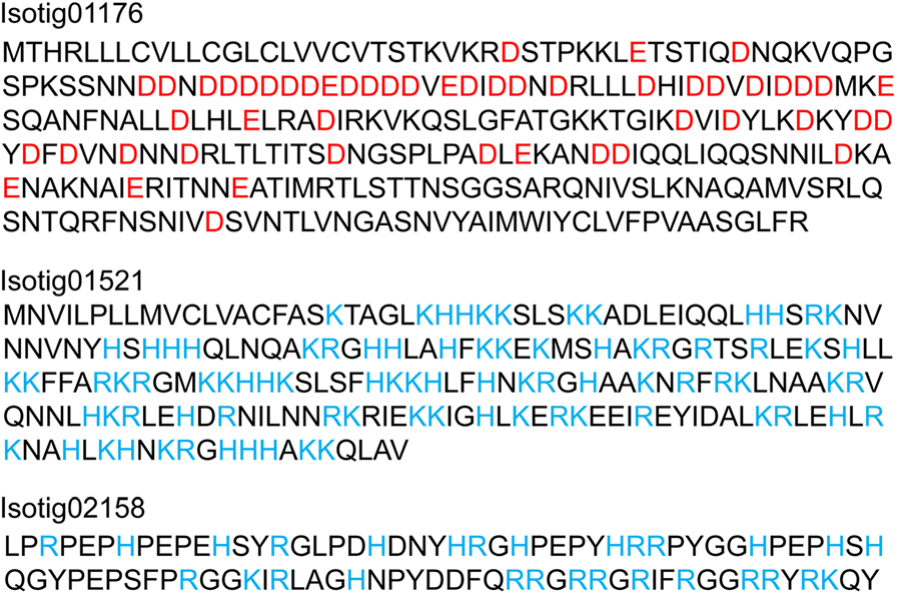
Amino acid sequences of isotig 01176, isotig 01521 and isotig 02158. Acidic amino acids are highlighted in red and basic amino acids are highlighted in blue

**Fig. 7:**
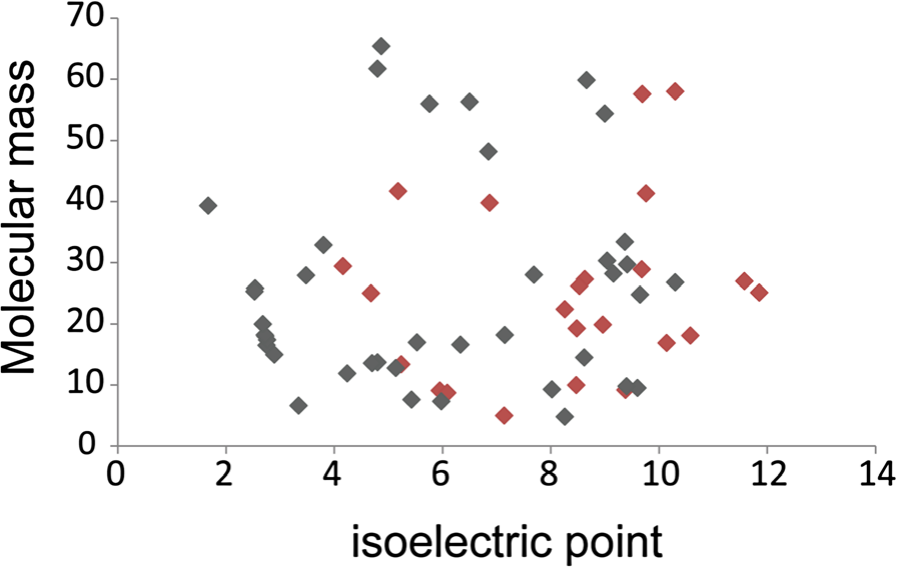
Molecular mass and isoelectric points. Red symbols represent shell matrix proteins from brachiopod and gray symbols represent shell matrix proteins from mollusca (Marin et al. 2008)

Shell matrix proteins of molluscs contain aspartic acid-rich proteins that are postulated to interact with Ca^2+^ [33]. Isotig 01176 has a repeat sequence comprising aspartic acid, suggesting that this region also binds Ca^2+^ (Fig. 6). However, the brachiopod shell matrix proteins identified in this study do not have unusually low isoelectric points seen for aspartic acid-rich proteins in the molluscan shell (Fig. 7). Among the shell matrix proteins identified in this study, isotig 01521 and isotig 02158 have a relatively high concentration of basic amino acids (Fig. 6). Although it is possible that unusually acidic shell matrix proteins have not been identified in this study, there may exist a general difference in isoelectric points between mollusc and brachiopod shell matrix proteins.

### Repeat sequences

**Fig. 8:**
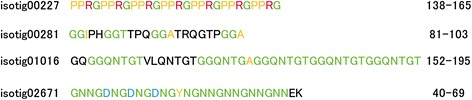
Repeat motifs found in shell matrix proteins identified in this study

Many of the shell matrix proteins identified in this study have repeat sequences, which are generally represented as a low complexity domain in the SMART prediction (Fig. 2). Repeat sequences exist in many skeletal proteins identified from other phyla and are thought to have important roles in biomineralization processes. The GXN (Glycine-X-Asparagine) repeat that is present in the amino acid sequence of isotig 02671 also exists in Nacrein, the carbonic anhydrase in the molluscan shell (Fig. 8). This repeat sequence has been proposed to function by inhibiting the precipitation of calcium carbonate [34]. Therefore, the GXN repeat in isotig 02671 could also be involved in the control of CaCO_3_ growth. Besides this repeat sequence, the repeat motifs PPRG, GGX, and GGQNTGX are also present in sequences of the shell matrix proteins (Fig. 8). Although the exact function of these repeat sequences is not clear, the existence of a variety of repeat sequences in skeletal proteins suggests that this sequence structure has fundamental roles in biomineralization.

### Possible mechanisms of brachiopod biomineralization

Many shell matrix proteins identified in this study have a signal peptide, suggesting that crystallization occurs in the outer cell space. This is consistent with shell matrix proteins in mollusca. Isotig 01176, which has an asparatic acid-rich region, NAD(P) binding domain, and ABC-type transport system, is a possible candidate that controls Ca^2+^ and H^+^ levels, whereas isotig 01521, which contains many basic amino acids, may interact with HCO_3_^−^. These proteins do not exist in skeletal proteins of other phylum, suggesting that the brachiopod has a unique ion control system for shell formation. In addition to these proteins, MSP130 and ICP-1 are thought to have important roles in shell formation.

### Evolution of brachiopod biomineralization

Skeletal proteins from brachiopods and other phyla share common structural features. The existence of MSP130 in brachiopod shell suggests that this protein have significant functions for biomineralization, and the several phyla share a part of biomineralization mechanisms. This protein could have been acquired by horizontal gene transfer from bacteria, but as discussed above, the data shown by Ettensohn (2014) are not conclusive, and MSP130 could also have been present in the last common ancestor of bilaterians or metazoans. On the other hand, proteinase and proteinase inhibitors did not show significant sequence similarities to the skeletal proteins in other phyla, suggesting that these proteins have been recruited as shell matrix proteins independently. In addition, almost all the brachiopod shell matrix proteins identified in this study had no homologous sequences in the public data banks, suggesting unique origins for those proteins. These results support the hypothesis that brachiopod biominerals were acquired independently from other phyla in the Cambrian explosion. On the other hand, there is also a possibility that brachiopod biominerals share the same ancestral biomineralization system with some other phyla, but after acquisition of biominerals, the matrix proteins evolved rapidly and/or novel lineage-specific proteins were added to the ancestral biomineralization system. The shell proteome of the mollusc *Lottia gigantea* showed that 25 out of 39 shell matrix proteins had no homologues, and only a few proteins showed high sequence similarity to shell matrix proteins in bivalves and skeletal proteins in other phyla [7]. This observation may well be reflecting the rapid nature of the evolution of shell matrix proteins, but can also be considered as reflecting the independent origin of the skeletal matrix proteins of molluscs from those of other phyla. Although more data are needed to address this problem, extremely low numbers of homologous shell matrix proteins between brachiopods and molluscs, combined with the presence of a possible unique ion control system involving basic shell matrix proteins, tend to support an independent origin for the brachiopod shells as expected from the phylogenetic relationships and the fossil record [1, 35].

## Conclusions

Our results identified two interesting shell matrix proteins, ICP-1 and MSP130. ICP-1 is a brachiopod shell matrix protein sequenced partially in previous studies, and MSP130 is a skeletal protein identified originally in sea urchins and oysters. Our data also showed novel shell proteins containing unique structures, including NAD(P) binding domains, suggesting the involvement of a hitherto unknown ion control system for shell formation. In addition, most other shell matrix proteins of *Laqueus rubellus* do not have a homologue in skeletal proteins of other phyla, suggesting an independent origin of the brachiopod shell. To further address the mechanisms and evolution of shell formation in brachiopods, additional studies, such as gene expression analysis and functional analysis, using this large-scale sequence information are necessary.

## Materials and methods

### RNA extraction and amplification

Live individuals of *Laqueus rubellu*s were collected by dredging operations in Sagami Bay, Japan (2 km off Jogashima, 90 m water depth). The mantle tissues from a single individual were separated from the shell using tweezers and homogenized in 500 μL of Isogen (Nippon Gene, Tokyo, Japan) in a 1.5-mL microcentrifuge tube. Total RNA was extracted following the manufacturer’s protocol and purified using the RNeasy Mini Kit (Qiagen, Hilden, Germany). Amplification of mRNA was performed using MessageAmp II aRNA Amplification Kit (Life Technologies, Carlsbad, CA, USA) to obtain sufficient quantities of mRNA (200 ng) for transcriptome analysis. The amount of the initial total RNA subjected to the amplification was 1 μg and the incubation time for in vitro transcription was 8 h.

### Transcriptome

The nucleotide sequences of the cDNA expressed in the mantle tissue were determined using 454 GS Junior (Roche, Basel, Switzerland). Template DNA was prepared according to the supplier’s protocol. A total of 125,437 reads of the GS Junior sequences were generated. The obtained reads were assembled using Newbler v.2.8. software with default settings, resulting in 2,342 isotigs.

### Extraction of shell matrix proteins

The shells were incubated overnight in a 5 % bleach solution with gentle shaking at room temperature to remove surface contaminants. After thorough washing with ultrapure water, the shells were crushed in water and dried. Organic materials were extracted by dissolution of the calcium carbonate using 0.5 M EDTA (pH 8.0) at a ratio of 23 mL to 1 g shell with shaking at 4 °C. After the solution was centrifuged at 20,000 *g* for 1 h, the insoluble organic materials separated by the centrifugation step were dissolved in an aqueous solution containing 9 M urea and 2 % (v/v) Triton X-100. The supernatant was concentrated and the EDTA removed using an Amicon Ultra-15 centrifugal filter unit with an Ultracel-3 membrane (Millipore, Billerica, CA, USA). The solution was then centrifuged at 15,000 *g* for 15 min and the supernatant was used for proteome analysis to identify the shell proteins in the soluble organic matrix. In addition to the matrix proteins of the whole shell, we also extracted proteins contained specifically in the shell secondary layer. The fibers of the shell secondary layer were collected by decantation after crushing the shells in water, and the soluble organic matrix was collected by the same method described above.

### SDS-PAGE analysis

To check the concentration and heterogeneity of the extracted proteins, the soluble organic matrix from the shell secondary layer was subjected to SDS-PAGE analysis using 4–20 % Mini-PROTEAN TGX Precast Gels (Bio-Rad, Hercules, CA, USA) with the standard method [36], and stained with CBB and silver staining.

### Peptide sample preparation by trypsin digestion

The soluble matrix proteins extracted with 0.5 M EDTA and the proteins that was insoluble in the first extraction step with EDTA and re-dissolved in solution containing 9 M urea and 2 % (v/v) triton X-100 were diluted in 0.1 M Tris–HCl (pH 8.5) to a final volume of 200 μL. Methanol (600 μL), chloroform (150 μL), and distilled water (450 μL) were added one-by-one and mixed thoroughly. After centrifugation at 12,000 rpm for 5 min at 4 °C, the upper aqueous layer was removed and 500 μL of methanol was added. After centrifugation at 15,000 rpm for 5 min at 4 °C, the supernatant was removed and the resultant precipitated proteins were dried using a Speed Vac for 2 min. Protein samples were re-dissolved in 10 μL of 8 M urea, 0.1 M Tris–HCl (pH 8.5), and mixed for 1 h. Subsequently, 0.5 μL of 0.1 M DTT was added and incubated at 37 °C for 1 h. Then, 0.5 μL of 208 mM iodoacetamide was added and incubated for 1 h in the dark. After adding 30 μL of 0.1 M Tris–HCl and 60 μL of ultra-pure water, sequencing grade modified trypsin (Promega, Fitchburg, WI, USA) was added, and the solution was incubated at 37 °C for over 15 h.

### LC-MS/MS analysis

The tryptic peptides were analyzed with a LTQ Orbitrap mass spectrometer (Thermo Fisher Scientific, Waltham, MA, USA) coupled with a DiNa nanoLC system (KYA Technologies, Tokyo, Japan). Precursor ions were detected over a range of 400–1,500 m/z, and the top four high-intensity ions were selected for MS/MS analyses in a data-dependent mode. Acquired MS/MS spectra were subjected to a database search against the protein sequence database translated from the transcriptome data from the mantle tissues of *Laqueus rubellus* with the SEQUEST program using Proteome Discoverer software version 1.2 (Thermo Fisher Scientific). The parameters was set as below: the charge state of the precursor ions: automatically recognized, the mass range of tryptic peptides: 800 to 4,500, mass tolerances for precursor ions: 10 ppm mass tolerances for fragment ions: 1 Da. Up to two missed cleavages and modifications of carbamidomethylation (+57.021) of cysteine and oxidation (+15.995) of methionine were considered for calculation of the theoretical masses. False discovery rates (FDRs) was calculated based on a decoy database and using the Proteome Discoverer software. A list of the identified peptides that include a <1 % false discovery rate was obtained after filtering low confidence identification.

### Sequence analyses

We performed BlastP similarity searches using the non-redundant protein sequence data stored in GenBank (http://blast.ncbi.nlm.nih.gov) using the default settings. The e-value cutoff was set at 1e-10. The domains in the protein sequences were predicted using the SMART program [37, 38] (http://smart.embl-heidelberg.de), including the optional searches for outlier homologues and homologues of known structure, Pfam domains and signal peptides, and the NCBI conserved domain search with the default settings [39–41] (http://www.ncbi.nlm.nih.gov/Structure/cdd/wrpsb.cgi). Isoelectric points and molecular masses of the predicted proteins were calculated using Genetyx version 6 (Genetyx, Tokyo, Japan). Sequence alignments were performed using Genetyx version 6.

### RT-PCR for gene expression analysis

The mantle and lophophore tissues from a single individual were homogenized in 500 μL of Isogen (Nippon Gene, Tokyo, Japan) in a 1.5-mL microcentrifuge tube. Total RNA was extracted following the manufacturer’s protocol, and treated with RQ1 RNase-Free DNase (Promega, Fitchburg, WI, USA). Reverse transcription was catalyzed by ReverTra Ace (Toyobo, Osaka, Japan), primed with a random primer. The amount of resultant cDNA was quantified by Qubit 2.0 Fluorometer (Life Technologies, Carlsbad, CA, USA), and an amount of 1 ng each of cDNA was used as template for polymerase chain reaction (PCR). PCR was catalyzed by Ex Taq Hot Start Version (Takara, Otsu, Japan). Partial sequence of ICP-1was amplified using the primer pair of Lr46S-1 (5’-GGC CAC ACC TCT GAT GGA TCA T) and Lr46A-1 (5’-TAC ACA CTT AAT GGA GAC CAG GC), and the annealing temperature was set at 58 °C. The following primer pairs were used for amplification of EF-1α; EF-B (5’-CCN CCD ATY TTR TAN ACR TCY TG) and EF-3 (5’-GGN CAY MGN GAY TTY RTN AAR AAY ATG AT), and the annealing temperature was set at 50 °C. Size and amount of RT-PCR products were verified by 1.5 % agarose gel electrophoresis.

## Additional files

Additional file 1:
**RT-PCR analysis of ICP-1 and EF-1α. M: mantle; L: lophophore.** (TIFF 897 kb) Additional file 2:
**The depth of the isotigs of shell matrix proteins in this study.** (TIFF 597 kb)
