# Supplementary figures and images for: Proteome analysis of shell matrix proteins in the brachiopod Laqueus rubellus
Source: Proteome Sci. 2015 Aug 15;13:21. doi: 10.1186/s12953-015-0077-2 (PMC4536745; doi:10.1186/s12953-015-0077-2)

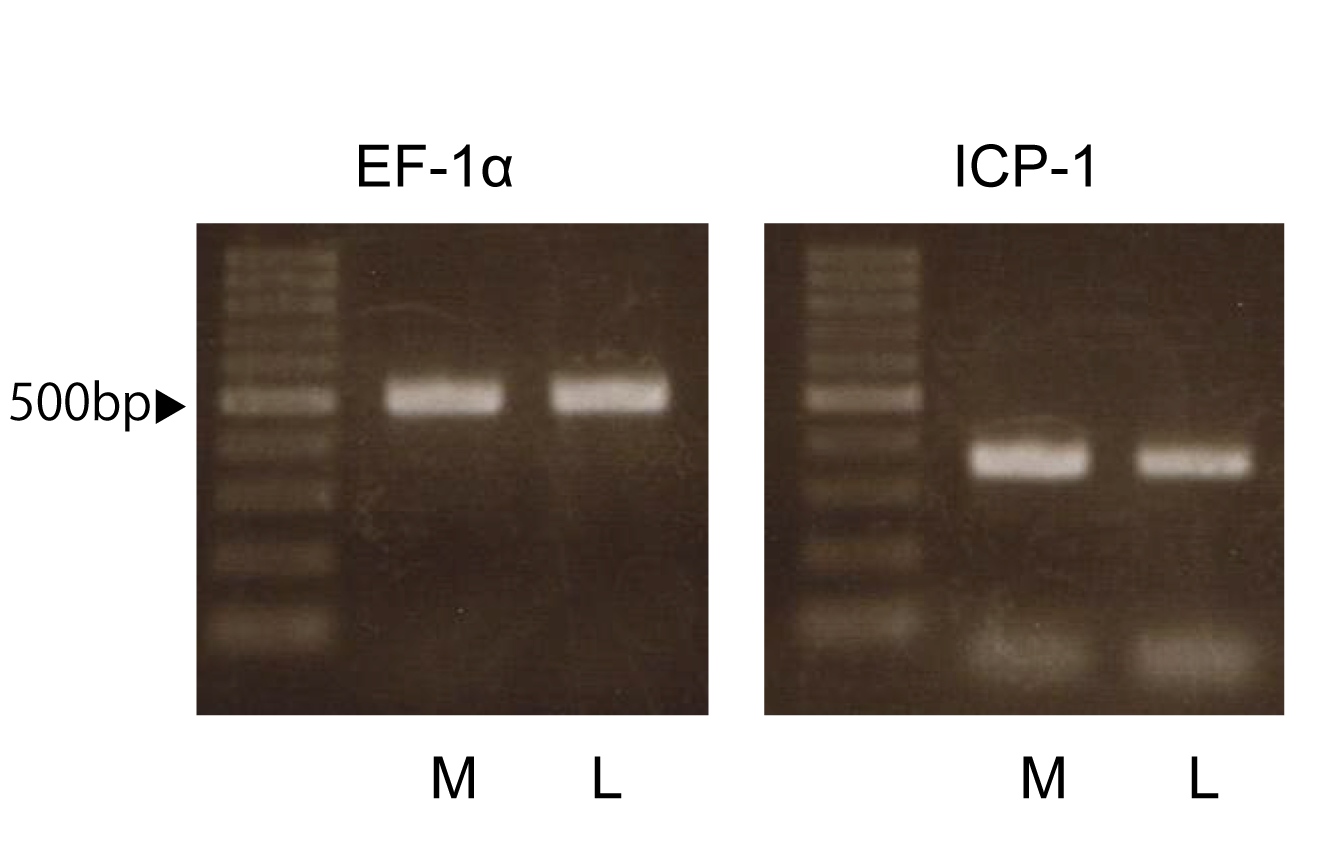

Supplement: Additional file 1: — RT-PCR analysis of ICP-1 and EF-1α. M: mantle; L: lophophore. (TIFF 897 kb) [file 12953_2015_77_MOESM1_ESM.tiff]

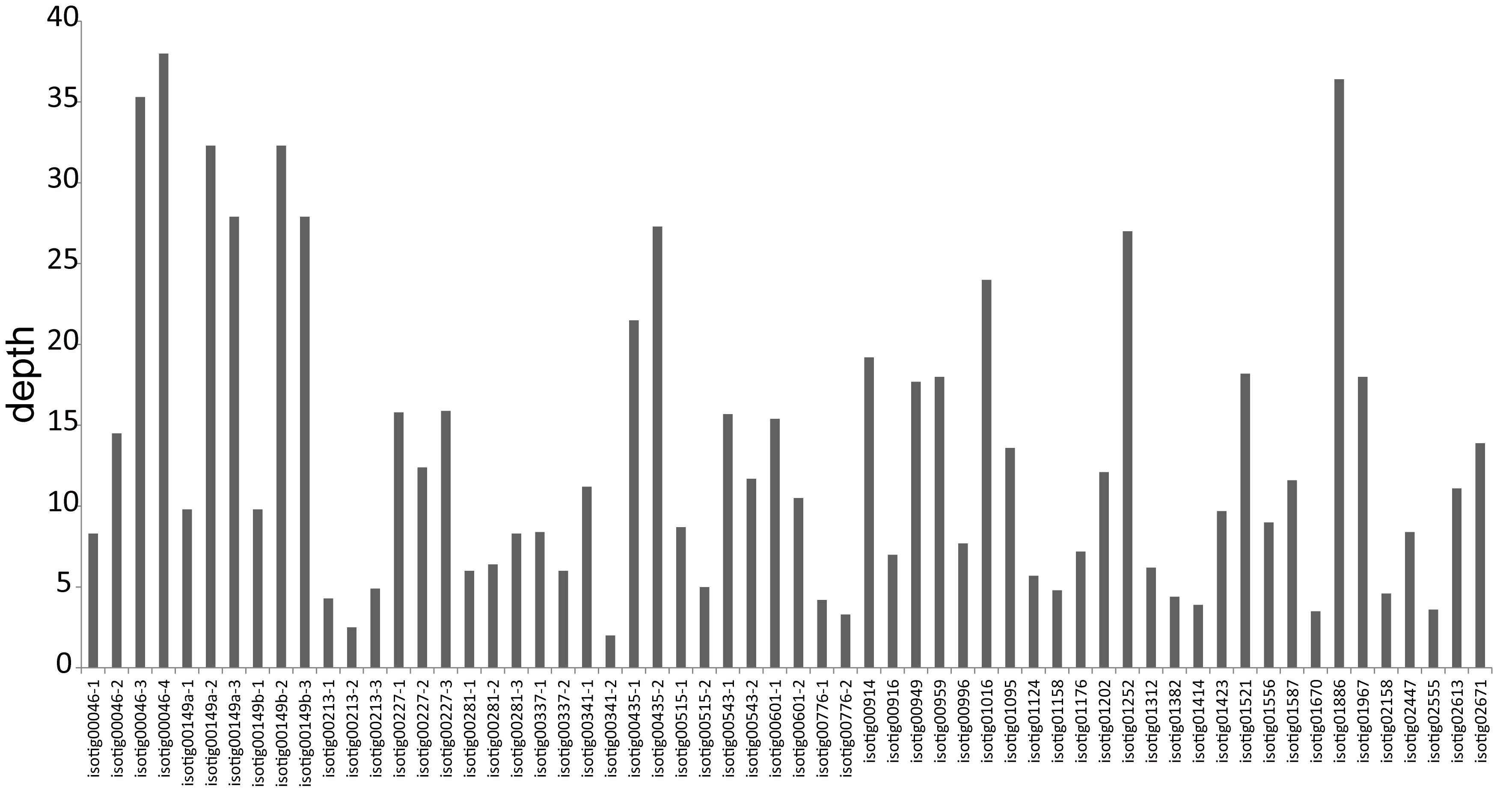

Supplement: Additional file 2: — The depth of the isotigs of shell matrix proteins in this study. (TIFF 597 kb) [file 12953_2015_77_MOESM2_ESM.tiff]
